# Modeling Impact of Land Use Dynamics on Hydrology and Sedimentation of Megech Dam Watershed, Ethiopia

**DOI:** 10.1155/2020/6530278

**Published:** 2020-10-24

**Authors:** Abebe Tarko Assfaw

**Affiliations:** Civil Engineering Department, College of Engineering, Debre Berhan University, P.O. Box 445, Debre Berhan, Ethiopia

## Abstract

Land use/land cover dynamics change the hydrology and sediment yield of the watershed. This research on how land use dynamics alters catchment hydrology and reservoir sedimentation aids the government to implement appropriate response strategies to minimize undesirable future impacts on the upper Megech dam reservoir. For this study, the impacts were quantified and analyzed through hydrological modeling (SWAT). The overall analysis was performed by using 1998 historical and 2016 recent land use satellite images. The analysis has shown that the cultivated land has increased from 60.69% to 67.17% and urban land from 2.3% to 3.36% between 1998 and 2016. Whereas the grassland area has decreased from 11.42% to 5.33%, plantation forest from 1.84% to 0.9%, and bareland from 3.58% to 2.56%. A comparison of the simulated outputs of the model shows that the mean annual surface runoff for 1998 land use was 251.3 mm and had changed to 316.7 mm in 2016 land use. The mean annual streamflow changed from 150.3 m^3^/sec to 165.6 m^3^/sec for 1998 and 2016 land uses, respectively. Similarly, 10.23 t/ha mean annual sediment load gets into Megech dam reservoir in 1998 LULC and was changed to 13.61 t/ha in 2016 LULC. This shows that streamflow, surface runoff, and sediment yield increases by 10.2%, 26.03%, and 33.3% in 2016 land use as compared with 1998 land use. Finally, the most dynamic subbasins that have a significant impact on streamflow and sediment yield were identified. Based on this, subbasins 13, 17, 19, 20, 23, 24, and 25 were found to be the most dynamic and change sensitive subbasins that have a significant contribution to the increment of runoff and sediment yield in Megech dam watershed.

## 1. Introduction

Natural and man-induced factors on global land cover change, especially in terms of change from forest cover to other land cover, has been one of the important issues on global change research. It is reported that more than 2/3 of farmland degradation in Africa is caused by soil erosion [1]. Rapid land use/cover change due to intensive agricultural practice in Ethiopian highlands results in increased rates of soil erosion and reservoir sedimentation [2]. Because of the rugged terrain, the rates of soil erosion and land degradation in Ethiopia are high [3]. According to Hurni, Ethiopia loses about 1.3 billion metric tons of fertile soil every year, and the degradation of land through soil erosion is increasing at a high rate. In the upper Blue Nile Basin, poor land use practices and lack of soil conservation strategies are major causes of downstream reservoir sedimentation. The land use of Megech dam watershed is changed rapidly due to the increasing demand for agricultural practice, urbanization, and deforestation. These call for immediate measures to safeguard the soil and water resources degradation of the country, specifically in Megech dam watershed.

Estimation of run off and sediment load is required in practical studies for the planning, design, operation, and maintenance of water resources structures [4, 5]. Different research studies [6–8] have been performed so far to estimate soil erosion in Ethiopian highlands. But the problem has been increasing and could be worse in the future due to the spatial and temporal heterogeneity in land use practice and soil properties. Due to the limited availability of relevant data and information, hydrological studies are often hindered in some highland areas of Ethiopia [9]. The main reasons for this are some of river basins are ungauged and unavailability of high-resolution spatial data such as the digital elevation model, soil properties, and land use data of the basins. Moreover, in gauged river basins, finding all the information essential for understanding the hydrological process is difficult due to the limited range of measurement techniques in space and time [10]. For such conditions, hydrological models provide an alternative solution. The purpose of using a model is to establish baseline characteristics whenever data are not available and to simulate long-term impacts such as land use dynamics that are difficult to calculate [11].

To understand the future effects of land use on streamflow and sediment yield, it is mandatory to know the effects historic land use changes have had on river flow and sediment production at a watershed level. Changes in land use and cover alter the runoff behavior, sediment yield, and the balance that exists between evaporation, groundwater recharge, and stream discharge in specific areas and in the entire watershed. Land use land cover changes are important driving forces for both runoff and sediment yield changes across all spatiotemporal scales. For example, the conversion of forest plantation to agricultural land between 1985 and 2011 periods in Angereb watershed in Ethiopia increased the runoff approximately by 39% [12]. It is also evident that land use/land cover changes also affect the sediment yield [13]. For instance, the expansion of cultivated land and vegetation cover loses in the Rib watershed of Ethiopia between 1986 and 2016 periods have increased the sediment yield significantly [14]. Effects of land use dynamics on hydrology and sediment production are the results of complex interactions between diverse site-specific factors and offsite conditions, and standardized types of responses will rarely be adequate. Though hydrologic response is an integrated indicator of watershed condition, land use dynamics possibly affects the overall characteristics of the watershed. Research of catchment hydrology and reservoir sedimentation under land use dynamics is a key to improve the management of the entire Nile Basin, specifically Megech dam watershed, so that it is possible to forecast the future effect of the land use change on the hydrology of the catchment. Therefore, data on how the catchment hydrology is changed concerning land use/land cover changes are necessary to improve the knowledge of catchment characteristics.

Estimates of long-term streamflow and sediment yield have been used for many decades to size the sediment storage pool and to estimate reservoir life. However, these estimates are often inaccurate, and many reservoirs have accumulated sediments more rapidly than originally planned. The primary possible cause for this can be ignorance of future trends such as the land use change impact. For instance, the Koka dam reservoir, in Ethiopia, lost its capacity at a rate of 2302 tons/km^2^/year over the last 30 years [15]. Hence, up-to-date knowledge of the sedimentation process and deposition would help in ensuring remedial measures to be taken in advance for optimum utilization of resources. Due to the reservoir sedimentation, Angereb dam constructed in early 1980, around the study area could not be serving up to the expected design period. Thus, the assessment of catchment hydrology and sediment deposition becomes very important for the management and operation of Megech reservoir, which is going to be constructed around the same area. Even though different research studies have been performed on the same catchment, land use dynamics impact on streamflow and sediment yield had not been evaluated yet. There is also a knowledge gap concerning the interdependence between land use dynamics and sediment production in the study area. All the above reasons imply that the study of land use dynamics impact on catchment hydrology and reservoir sedimentation for Megech dam watershed is required. Hence, a proper investigation of the sediment and runoff yield of the catchment at different land use/land cover data has been performed for the management of sedimentation and utilization of water resources.

The objective of the research was to model the impact of land use dynamics on catchment hydrology and reservoir sedimentation. The research was also intended to evaluate the SWAT model capability in predicting the spatial variability of runoff and sediment yield from Megech dam watershed. Besides, the research was deliberately performed to identify and prioritize the most dynamic subbasins that are sensitive to land use change in the watershed.

### 1.1. Study Area

Megech dam watershed is located at a distance of 725 km northwest of Addis Ababa. It is one of the Lake Tana subbasin catchments mainly situated in Gondar Zuria of the Amhara region.

**Figure 1:**
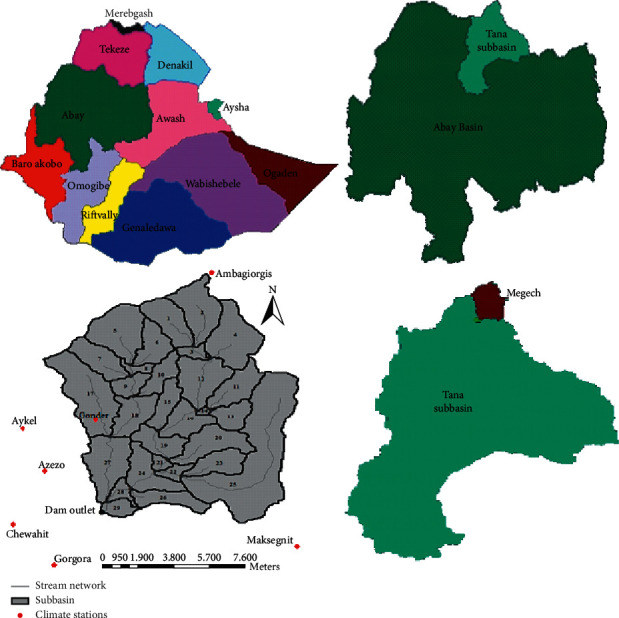
Location map of upper Megech dam watershed.

The catchment area at the dam site is 39,419.2 ha, which is fully gauged. The geographical location of the study area is between 37°18′14″ and 37°37′19″ east longitude and 12°16′25″ and 12°45′27″ north latitude. The location map of Megech dam watershed is shown in Figure 1. The Megech river, which flows generally in a southern direction and empties into Lake Tana, is one of the main streams flowing into Lake Tana from the north. Angereb, Keha Eyesus, Guara, and Argif rivers are the major tributaries that join Megech River. The mean annual precipitation is about 1,050 mm with 1,100 mm in the upper part and about 1,000 mm in the lower part. Temperature varies from 11.5°C to 30°C. Maximum temperatures vary from 23°C in July to 30°C in March, whereas minimum temperatures range from 11.5°C in January to 15.6°C in April and May. Humidity varies between 39% in March and 79% in August. Wind speed is low, thus minimizing potential evapotranspiration values between 101 mm/month in July and 149 mm/month in March [16].

The highest elevation of the Megech dam watershed is 2953 m above mean sea level, in its northeastern part. The lowest topography land is at the dam site, which is at an altitude of 1878 m. Flat, undulating plains, rolling plains, hilly plains, and mountainous landforms are the major topographic features of the Megech dam watershed [17]. The catchment landform of Megech dam watershed is characterized as a mountainous, wedge-shaped, and steep-sloped.

Megech dam reservoir capacity is about 182 M m^3^ of water. The dam axis is located in between the geographic grid reference UTM E 332995, N1382164 and E332492, N1382864. The location of the riverbed at the center of the dam axis (in UTM) is *E* = 332646 m and *N* = 1382648 m with an elevation of 1877 m. The dam is currently under the construction phase, and it is rock fill embankment type with a crest length of 890 m and height of 76.5 m above the riverbed level. The dam would allow developing around 7,311 hectares of land using irrigation, besides securing water demand for Gondar town. The dam site is characterized by broad and flat flood plains, old bench forming terrace, and low to high relief basaltic hills with steep to moderately steep slopes. The right abutment of the dam is characterized by a steeper slope with a slope angle of 28°, whereas the left abutment is of the milder slope with a slope angle of 8° [16].

**Figure 2:**
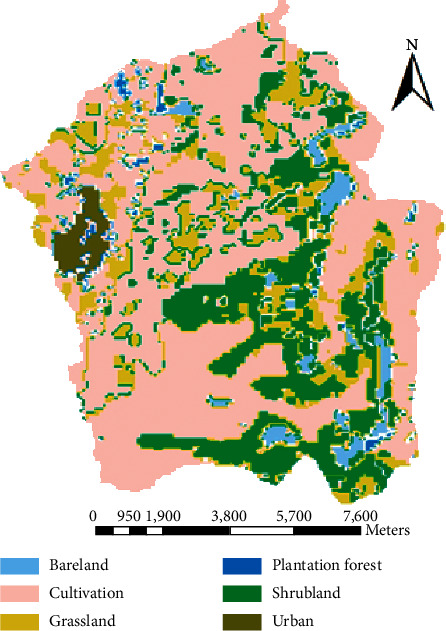
Land use map of the study area.

**Figure 3:**
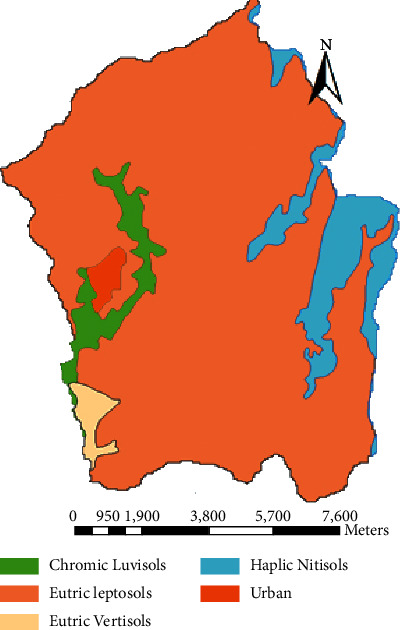
Soil map of Megech dam watershed.

The Megech dam watershed is presently covered with six types of land covers, namely, bareland, cultivated land, grassland, shrubland, urban, and plantation forest (Figure 2). Cultivated land covers the highest portion of the watershed. According to the FAO soil classification system, the major and dominant soils identified in the Megech dam watershed are Eutric Leptosols, Eutric Vertisols, Urban, Chromic Luvisols, and Haplic Nitosols. As could be seen from the map (Figure 3), most of the watershed area is covered with Eutric Leptosols.

## 2. Materials and Methods

**Figure 4:**
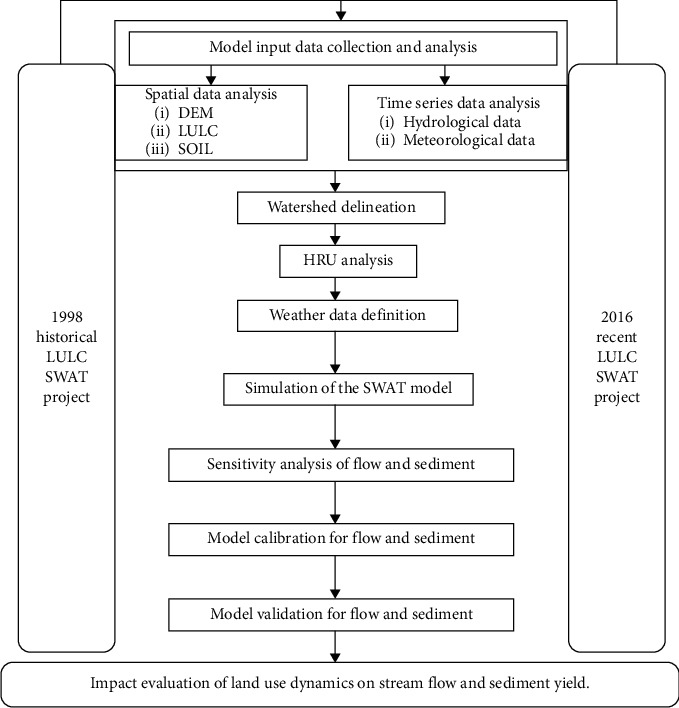
Conceptual framework of the research methodology.

The overall input data preparation, analysis, and modeling were performed by using the Arc GIS software and its extension, the Arc SWAT model. In general, two types of data, namely, spatial and time series data, were collected and used for this study. The data collected were processed until they become an input to the model used. First, spatial data (DEM, soil map, and land use map) were defined using Arc GIS with the same projection. At this step, watershed delineation and hydraulic response unit analysis were performed for the spatial input data. Second, the weather data table and gauging location for the selected weather stations and synoptic/weather generator station were prepared in the required format of the model (dbf). For the weather generator/synoptic station, all the required values were computed both manually and using helping softwares such as WGNmaker4.xlsm and dew02.exe program. Once the model is parameterized by converting the results of data analysis into model parameters, then model sensitivity analysis, calibration, validation, and simulation for different land use changing were conducted and end up with some conclusions and recommendations.  Figure 4 shows the overall conceptual framework of the research methodology.

### 2.1. Meteorological Data

There are six meteorological observation stations within and around the Megech watershed, namely, Aykel, Gondar, Ambagiorgis, Chewahit, Gorgora, and Maksegnit. For each gauging station, the required daily meteorological data (daily precipitation, daily maximum, and daily minimum air temperature, daily solar radiation, daily wind speed, and daily relative humidity) were collected from the National Meteorological Agency of Ethiopia (NMA) from 2004 to 2018. Rain gauges represent point sampling of the areal distribution of a storm. In practice, hydrological analysis requires knowledge of the rainfall over an area. Arithmetic mean, Thiessen polygon, isohyetal methods are some of the methods used to convert point (gauged) rainfall values at various stations into an average value over a catchment. However, Thiessen polygon is used for this study due to its simplicity, and the average rainfall over the catchment is calculated by the following equation:

(1) $$\begin{array}{c} P_{\text{ave}}=\overset{n}{\underset{i=1}{\sum }}\left(\frac{P_{i}*A_{i}}{A_{t}}\right), \end{array}$$

where *P*_ave_ is the average areal rainfall (mm), *P*_1_, *P*_2_, *P*_3_,…, *P*_*n*_ is the precipitation of stations 1, 2, 3,…, *n*, respectively, and *A*_1_, *A*_2_, *A*_3_,…, *A*_*n*_ is the area coverage of stations 1, 2, 3,…, *n*, respectively, in the Thiessen polygon.  Table 1 shows the area weight of all rainfall stations.

Checking the availability, quality, consistency, and homogeneity of hydrometeorological data is imperative for any hydrological model study such as SWAT. Hence, to simulate a hydrological system, both the climatic and hydrological data should be stationary, consistent, and homogeneous when they are used for frequency analysis. Therefore, to determine whether the data collected meet these criteria, we need to have an efficient screening procedure. For this study detail, discussion on rainfall, streamflow, and sediment yield data analysis was performed. Due to its low impact on SWAT application, only a simple graphical plot and visual examination were made for the remaining meteorological data.

### 2.2. Filling Missing Rainfall Data

For this study, both the normal ratio method and the arithmetic mean method were used to fill the missing rainfall data. The normal ratio method was used when the mean monthly rainfall of one or more of the adjacent (index) stations differs from that of the missed record station by more than 10% (equation (2)).

(2) $$\begin{array}{c} P_{x}=\frac{1}{N}\left(\frac{N_{x}}{N_{A}}P_{A}+\frac{N_{x}}{N_{B}}P_{B}+\frac{N_{x}}{N_{C}}P_{C}+\cdots +\frac{N_{x}}{N_{N}}P_{N}\right), \end{array}$$

whereas the simple arithmetic mean method was used where the mean monthly rainfall of all the index stations is within 10% of the station under consideration (equation (3)).

(3) $$\begin{array}{c} P_{x}=\frac{1}{N}\left(P_{A}+P_{B}+P_{C}+\cdots +P_{N}\right), \end{array}$$

where *N* is the number of index stations, *Px* is the precipitation for the station with the missed record, *P*_*A*_+*P*_*B*_+*P*_*C*_+⋯+*P*_*N*_ is the corresponding precipitation at the index stations, and *N*_*A*_+*N*_*B*_+*N*_*C*_+⋯+*N*_*N*_ and *Nx* are the long terms mean annual precipitation at the index stations and at station *x* under consideration, respectively.

### 2.3. Homogeneity Test

For this study, homogeneity of the selected rainfall stations had been checked by using nondimensional values of the monthly precipitation. This nondimensional value of the monthly precipitation was calculated by taking 15 years average value for each station. All stations' nondimensional values of the monthly precipitation were plotted together to compare the station's homogeneity with each other. Nondimensional values of the monthly precipitation of each station were computed by using the following equation:

(4) $$\begin{array}{c} P_{i}=\left(\frac{P_{i,av}}{P_{av}}\right)*100, \end{array}$$

where *P*_*i*_ is the nondimensional value of precipitation for the month in station *i*, *P*_*i*,*av*_ is the over years (15 years) averaged monthly precipitation for the station *i*, and *P*_*av*_ is the over year's averaged yearly precipitation of the station *i*.

### 2.4. Consistency Test

In this study, consistency of rainfall data was checked by using the double mass curve analysis method for the research period (2004–2018). Double mass curve analysis is a graphical method for identifying and adjusting inconsistency in a station record by comparing its time trend with those of adjacent stations. The method was used by plotting the cumulative mean annual rainfall of all stations versus the cumulative annual rainfall of each station separately. For the rainfall data to be consistent spatially, characteristics of the long-term record should not be subjected to a significant change with time. If the conditions relevant to the recording of a rain gauge station have undergone a significant change during the period of record, inconsistency would arise in the rainfall data of that station. This inconsistency can be differentiated from the time of which significant change took place. If a significant change in the slope of the double mass curve is observed, it should be corrected by using the following equation:

(5) $$\begin{array}{c} P_{cx}=P_{x}*\frac{M_{c}}{M_{a}}, \end{array}$$

where *P*_*cx*_ is the corrected precipitation at any period, *P*_*x*_ has the originally recorded precipitation at period, *M*_*c*_ is the corrected slope of the double mass curve, and *M*_*a*_ is the original slope of the double mass curve.

### 2.5. Spatial Data

The Landsat images for spatial data, namely, the digital elevation model (DEM), soil map, and land use/land cover map, were collected from the Ministry of Agriculture (MoA) and Ministry of Water, Irrigation, and Energy (MoWIE).

**Figure 5:**
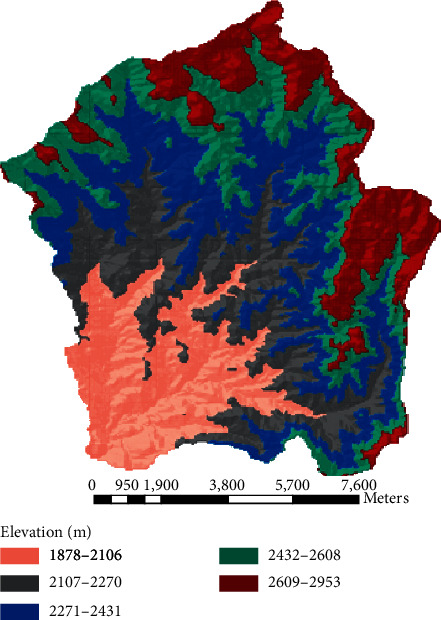
Elevation map of the watershed.

**Figure 6:**
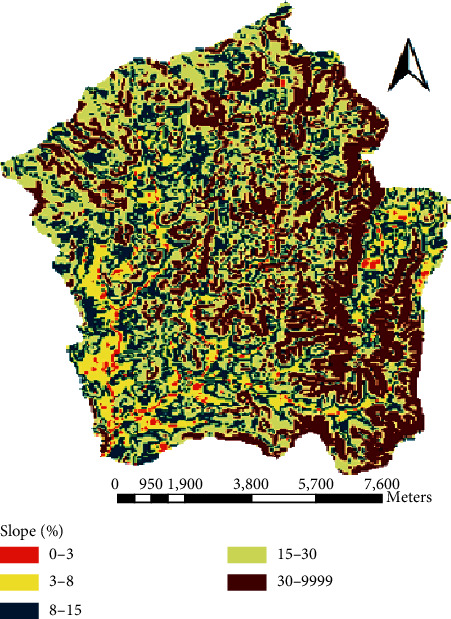
Slope classes of Megech dam watershed.

The digital elevation model **(**DEM) was collected from the Ministry of Water, Irrigation, and Energy (MoWIE). The elevation of the study area ranges from 1878 m to 2953 m a.s.l. The table shows the area coverage of different landforms in the watershed. Figures 5 and 6 show the elevation and slope classification of Megech dam watershed, respectively (Table 2).

The soil shapefile, which describes the distribution of soil in the study area, was obtained from the Ministry of Water Resources, Irrigation, and Energy. It was observed that Eutric Leptosols, Haplic Nitisols, Chromic Luvisols, Eutric Vertisols, and Urban types of soils are the most dominant soils in the catchment as shown in Table 3.

The soil textural and physicochemical properties required by the SWAT model such as soil texture, available water content, hydraulic conductivity, bulk density, and organic carbon content for each soil type were obtained from FAO.

**Figure 7:**
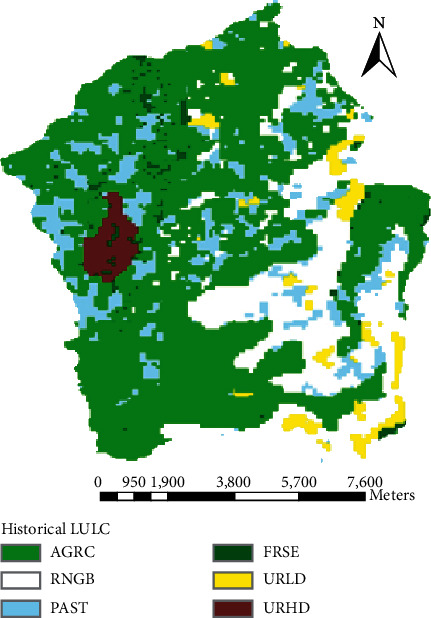
Historical land use (1998 LULC map).

**Figure 8:**
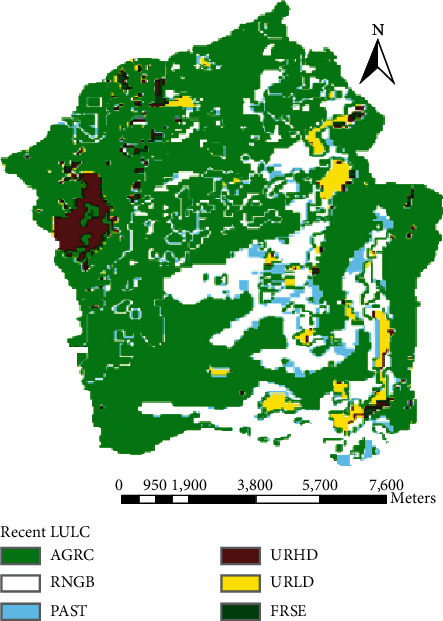
Recent land use (2016 LULC map).

To model, the impact of land use dynamics on catchment hydrology and sediment yield two reference land use maps with 18 years gap were considered. It was possible to get land use/land cover maps from satellite images for years 1984, 1998, 2008, and 2016. But all the maps were not having the same scale, spatial resolution, and season of map preparation. Only 1998 and 2016 LULC maps have the same scale, spatial resolution, and season of map preparation. The scale of the two maps was 1 : 50,000, and their season of map preparation was in dry season, whereas their spatial resolution was 90 m*∗*90 m. Due to the above reasons, only two land use maps (the 1998 historical LULC and 2016 recent LULC) were selected based on their correspondence in the scale, spatial resolution, and season of map preparation. The source of satellite images for both land use maps were the Ministry of Agriculture and Rural Development and Rural Land Management Directorate. Figures 7 and 8 show the spatial distribution and extent of land use dynamics from 1998 to 2016, respectively. Besides, Table 4 provides a summarized comparison on how the land use dynamics has taken place between the two land use phases (historical and recent).

### 2.6. Hydrological Data

The daily flow data were collected from the Ministry of Water, Irrigation, and Energy (MoWIE) and Hydrology and Water Quality Directorate for the period of 2004–2018 (starting on Jan 1–2004) at Azezo gauging station. Megech River has only one streamflow gauge around the Megech dam site near Azezo. Hence, filling the missing data was made by making relations within the data of the gauge itself instead of making related to the other stations outside the Megech River. Even though modest rains are experienced in other seasons, wet seasons (June–September) are the main source of streamflow in Megech River. There are two known methods commonly used for the infilling of the omitted streamflow data. The first one is by using linear regression between consecutive wet season months, which is recommended for the only wet season. Whereas, in the second method, the recession curve method is suggested for the dry season and can be calculated by the following equation:

(6) $$\begin{array}{c} Q_{t}=Q_{to}\exp\left(-\frac{t-t_{o}}{k}\right), \end{array}$$

where *Q*_*t*_ is the missed flow data (m^3^/s) in a day, *Q*_*to*_ is a specified initial daily mean discharge (m^3^/s), and *k* is the watershed characteristics, and it is the inverse of flow recession (*α*) also called a reaction factor. *k* can be calculated by the slope of the logarithmically transformed values of the flow last before the gap at time *t*_0_ (Q_*t*0_) and the first flow value after the gap at time *t*_1_ (*Q*_*t*1_) using the following equation:

(7) $$\begin{array}{c} \frac{1}{k}=\frac{\ln\;Q_{to}-\ln\;Q_{t1}}{t_{1}-t_{o}}. \end{array}$$

Noncontinuous-time step suspended sediment measurement was taken by the MoWIE and Hydrology and Water Quality Directorate near Azezo gauging station. Due to the lack of continuous-time step suspended sediment records, the sediment rating curve was developed for this particular study by using the measured sediment records as a function of the corresponding streamflow values. The sediment rating curve is a widely applicable technique for estimating the suspended sediment load being transported by a river through signifying a relationship between the stream discharge and sediment concentration or load [18]. The general relationship of the suspended sediment rating curve can be written as in the following equation:

(8) $$\begin{array}{c} Qs=a*Q^{b}, \end{array}$$

where *Qs* is the sediment load in ton/day, *Q* is the stream discharge in m^3^/s, and *a* and *b* are the regression constants. To work on the above formula, the first task was the conversion of the measured suspended sediment concentration (mg/l unit) records that were collected from the MoWIE into sediment load (ton/day unit) by using the following equation:

(9) $$\begin{array}{c} S=0.0864\times Q\times C, \end{array}$$

where *S* is the sediment load in (ton/day), *Q* is the stream flow (m^3^/s), *C* is the sediment concentration (mg/l), and 0.0864 is the conversion factor.

After calculating the sediment load in ton/day unit, the next step was making the relation between the continuous (daily time step) measured flow in m^3^/s and the measured sediment load (ton/day).

### 2.7. SWAT Model Setup and Input Parameterization

SWAT model is a semidistributed physical model that could best predict hydrology and sediment yield under land use dynamics [19]. SWAT simulates the hydrological cycle based on the water balance equation [20] and computed by the following equation:

(10) $$\begin{array}{c} SW_{t}=SW_{o}+\sum_{i=1}^{t}\left(R_{\text{day}}-Q_{\text{surf}}-E_{\text{a}}-W_{\text{seep}}-Q_{\text{gw}}\right), \end{array}$$

where *SW*_*t*_ is the final soil water content (mm H_2_O), SW_*o*_ is the initial soil water content on day *i* (mm), *t* is the time (days), *R*_day_ is the amount of precipitation on day *i* (mm H_2_O), *Q*_surf_ is the amount of surface runoff on day *i* (mm H_2_O), *W*_seep_ is the amount of water entering vadose zone from the soil profile on day *i* (mm H_2_O), *E*_a_ is the amount of evapotranspiration on day *i* (mm H_2_O), and *Q*_gw_ is the amount of return flow on day *i* (mm H_2_O).

According to William, 1996 sediment yield due to water erosion in the SWAT model is estimated using the following Modified Universal Soil Loss Equation (MUSLE):

(11) $$\begin{array}{c} S_{\text{ed}}=11.8{\left(Q_{\text{Surf}}\cdot q_{\text{Peak}}\cdot {\text{area}}_{\text{hru}}\right)}^{0.56}\cdot K_{\text{USLE}}\cdot C_{\text{USLE}}\cdot P_{\text{USLE}}\cdot LS_{\text{USLE}}\cdot \text{CFGR}, \end{array}$$

where *S*_ed_ is the sediment yield on a given day (metric tons), *Q*_Surf_ is the surface runoff volume (mm of water/ha), *q*_Peak_ is the peak runoff rate (m^3^/s), area_hru_ is the area of the hydrological response unit (ha), *K*_USLE_ is the USLE soil erodibility factor, *C*_USLE_ is the USLE cover and management factor, *P*_USLE_ is the USLE support practice factor, *LS*_USLE_ is the USLE topographic factor, and CFRG is the coarse fragment factor.

The SWAT model processes the digital elevation model (DEM), mapped land use, and soil data to create a set of default model input files. The first task in the SWAT model setup for watershed simulation is DEM-based stream and watershed delineation. The center of Megech dam axis at the riverbed level was taken as an outlet point for watershed delineation. The stream network was defined by the model using the concept of flow direction and flow accumulation. SWAT works on a subbasin level. The subdivision of a watershed into discrete subwatershed areas enables the modeling process to characterize the heterogeneity of the watershed. The size of subbasin in the watershed will affect the assumption of homogeneity. Hence, the definition of the watershed, subbasin boundaries, and streams depends on a threshold area, and this threshold area defines the minimum drainage area required to form the origin of a stream [21]. Configuration of a lot of subbasin requires a long time simulation period and even difficult to run. On the other hand, a small number of subwatersheds could affect the simulation results by ignoring spatial variability and lumps watershed conditions together. Based on this, the watershed was divided into 29 subbasins with a total of 944 grid cells, and the model automatically delineates a watershed area of 39419.2 ha.

Once the stream and watershed delineation has been accomplished, the definition of HRU was the next step of the SWAT model setup. Hydrologic response units (HRUs) are lumped land areas within the subbasin that comprised of unique land cover, soil, and slope combinations. HRUs enable the model to reflect differences in evapotranspiration, precipitation, runoff, infiltration, and other hydrologic conditions for different land covers, soils, and slope. The runoff and sediment yield is estimated separately for each HRU and routed to obtain the total value for the watershed. This increases the accuracy in streamflow and sediment yield prediction. Taking the research goal (land use dynamics impact) and recommendations into consideration, 5%, 15%, and 20% threshold levels for the land use, soil, and slope were applied, respectively, in HRU definitions to encompass most of the spatial details.

### 2.8. Sensitivity Analysis, Calibration, and Validation

The default simulation output in the SWAT model run cannot be directly used for further analysis. Instead, the ability of the model to sufficiently predict the constituent stream flow and sediment yield should be evaluated through sensitivity analysis, model calibration, and model validation [22]. Performing a calibration process for all model parameters becomes complex and computationally far-reaching [23]. In such cases, sensitivity analysis is helpful to identify rank parameters that have a significant impact on specific model outputs of interest [24]. For this study, the sensitivity analysis was performed using SWAT interface for a period of January 1, 2006 to December 31, 2012, in which the first two years (2004 and 2005) were taken as a warm-up period. After running sensitivity analysis, the sensitive parameters were categorized into four classes based on their mean relative sensitivity (MRS). The four classes are small to negligible (0 ≤ MRS < 0.05), medium (0.05 ≤ MRS < 0.2), high (0.2 ≤ MRS < 1), and very high (MRS ≥ 1) [11]. Based on this classification, both flow and sediment parameters with mean relative sensitivity value of medium to very high had been selected for calibration.

When a SWAT simulation of Megech dam watershed has taken place, there was a discrepancy between measured data and simulated outputs. Therefore, a combination of the automatic and manual calibration was performed until the simulated result matches the observed data. Calibration of streamflow and sediment yield was carried out at the outlet of subbasin 29 (near Azezo gauging station). This site was selected due to the availability of measured flow and sediment data. The streamflow and sediment calibration were on annual and monthly average time steps. For each calibration run and parameter change, the corresponding model performance statistics (*R*^2^ and ENS) were calculated. This procedure continued until the acceptable calibration statics recommended by the SWAT developer for hydrology was achieved. SWAT developers assumed an acceptable calibration for hydrology at *R*^2^ > 0.6 and ENS > 0.5 [25]. Generally, the model calibration and validation periods for both land use data years (LULC 1998 and LULC 2016) were selected as shown in Table 5 based on the available model input data.

The first two years of the calibration period (2004 and 2005) were used as model warm-up periods and were not used for model evaluation.

Model validation is testing of calibrated model results with independent dataset without any further adjustment at different spatial and temporal scales [20]. It is a process of proving the performance of the model. Validation is carried out for periods different from the calibration period but without any further adjustment of calibrated parameters. Both statistical model performance measures (regression coefficient and Nash and Sutcliffe simulation efficiency) used in the calibration procedure were also used in validating streamflow and sediment yield. Three methods for performance evaluation of model predictions were used during the calibration and validation periods. These numerical model performance measures are regression coefficient (*R*^2^), Nash and Sutcliffe simulation efficiency (ENS), and relative volume error (RVE) [26].

Regression coefficient is the square of the Pearson product-moment correlation coefficient and describes the proportion of the total variance in the observed data that can be explained by the model. The closer the value of *R*^2^ to 1, the higher is the agreement between the simulated and the measured flows. The range of values for *R*^2^ is between 1, best to 0, poor. *R*^2^ value greater than 0.6 is acceptable [25], and its value can be calculated by the following equation:

(12) $$\begin{array}{c} R^{2}=\frac{{\left[\sum_{i=1}^{n}\left(q_{si}-q_{s}\right)\left(q_{oi}-q_{o}\right)\right]}^{2}}{\sum_{i=1}^{n}{\left(q_{si}-q_{s}\right)}^{2}\sum_{i=1}^{n}{\left(q_{oi}-q_{o}\right)}^{2}}, \end{array}$$

where *q*_*si*_ is the simulated values of the quantity in each model time step, *q*_*oi*_ is the measured values of the quantity in each model time step, *q*_*s*_ is the average simulated value of the quantity in each model time step, and *q*_*o*_ is the average measured value of the quantity in each model time step.

Nash and Sutcliffe simulation efficiency (ENS) value greater than 0.5 is acceptable [25] and can be calculated using the following equation:

(13) $$\begin{array}{c} \text{ENS}=1-\frac{\sum_{i=1}^{n}{\left(q_{oi}-q_{si}\right)}^{2}}{\sum_{i=1}^{n}{\left(q_{oi}-q_{o}\right)}^{2}}, \end{array}$$

where *q*_*si*_ is the simulated values of the quantity in each model time step, *q*_*oi*_ is the measured values of the quantity in each model time step, and *q*_*o*_ is the average measured value of the quantity in each model time step.

Relative volume error (RVE) can vary between ∞ and −∞ but performs best when a value of 0 is generated, since no difference between simulated and observed discharge occurs. A relative volume error of less than +5% or −5% indicates that a model performs well, while relative volume errors between +5% and +10% and –5% and −10% indicate a model with a reasonable performance. It can be calculated using the following equation:

(14) $$\begin{array}{c} \text{RVE}=\frac{\sum \left(Q_{\text{sim}}-Q_{\text{obs}}\right)}{\sum Q_{\text{obs}}}*100\%, \end{array}$$

where RVE is the relative volume error in %, *Q*_sim_ is the simulated discharge, and *Q*_obs_ is the observed discharge in each model time step.

## 3. Results and Discussion

### 3.1. Rainfall Data Analysis Result

**Figure 9:**
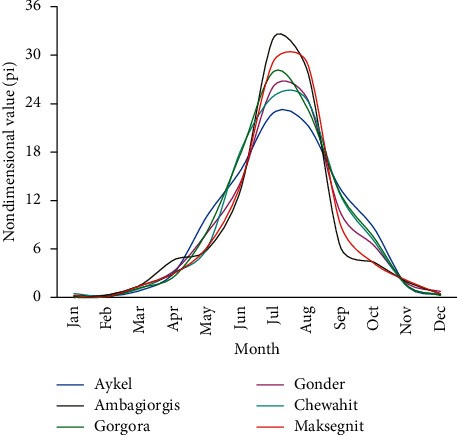
Homogeneity test plot of selected stations.

**Figure 10:**
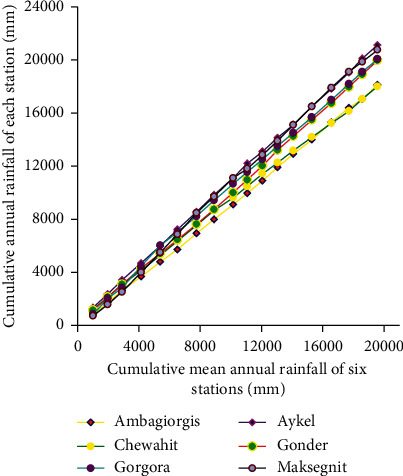
Double mass curve consistency test plot.

Four nonclass 1 stations, namely, Ambagiorgis, Gorgora, Chewahit, and Maksegnit, had a lot of missed rainfall data, while Gondar and Aykel (class-1 station) had somewhat good quality data with modest missing records. For this study, missing values were estimated from other stations around the missed record station by using both the normal ratio method and simple arithmetic mean method. After filling the missed rainfall values in all stations, both the homogeneity and consistency tests were performed. The nondimensional homogeneity test plot shown in Figure 9 confirms that all of the rainfall stations used for this particular study was homogeneous, and their rainfall pattern was found to be monomodal with high rainfall season from July to September and low rainfall season from February to March. Whereas the double mass curve spatial consistency test plot shown in Figure 10 indicates that Chewahit and Maksegnit stations data had a break. This is a sign of inconsistency for the Chewahit and Maksegnit station data. This inconsistency was corrected by adjusting the slope of the double mass curve using equation (5) before using the data as an input to the model.

### 3.2. Spatial Data Analysis Result

As the historical land use/cover map shows that the upper Megech dam watershed is covered by 60.69% of cultivated land, 1.84% of plantation forest, 11.42% of grassland, 20.17% of shrubland, 3.58% of bareland, and 2.30% of urban. The catchment is dominantly covered by cultivated land. The 1998 historical reference land use/land cover map shows that most of the southern, northern, and central parts of the watershed area were covered with cultivated land.

The recent 2016 land use/land cover map shows that the catchment is covered by 67.19% of cultivated land, 0.9% of plantation forest, 5.33% of grassland, 20.68% of shrubland, 2.56% of bareland, and 3.36% of urban. In this recent land use map, the plantation forest, grassland, and bareland were reduced and replaced by cultivated land and urbanization. Comparing to the 1998 LULC, the cultivated land coverage is increased by 6.48% in the 2016 LULC map. This was a good indicator of the expansion of the intensive agricultural practice in the watershed, which was caused by population growth in the area. At the same time, the plantation forest is reduced by 0.94%. This indicates that how deforestation is expanded in the area and forests are changed to cultivated land for food security purposes. Also, 6.09% of grassland and 1.02% of bareland are changed to urban and cultivated land, which is also an indication of greater cultivation land and settlement area demand in the catchment. A comparison of the two reference maps shows that greater change has taken place on cultivated land, which is an increment of 6.48% within 18 years gap (1998–2016). All the spatial analysis results of the land use dynamics could be summarized in Table 6.

### 3.3. Flow and Sediment Data Analysis Result

Visual examination of daily streamflow records for Megech River shows a good quality serial correlation with only a few missing data. For this study, the linear regression method was applied to fill the missing data since all the missing data were observed only during wet seasons. Unlike streamflow data, sediment data records exhibit several jumps. From the rating curve plot, the relationship between the flow and sediment load with *R*^2^ = 86.81% was found as shown in the following equationfd15:

(15) $$\begin{array}{c} Q_{s}=29.11*Q^{1.2301}, \end{array}$$

where *Q* is the streamflow (m^3^/s), and *Q*_*s*_ is the suspended sediment load (t/day). The relationship is known as a suspended sediment rating curve.

**Figure 11:**
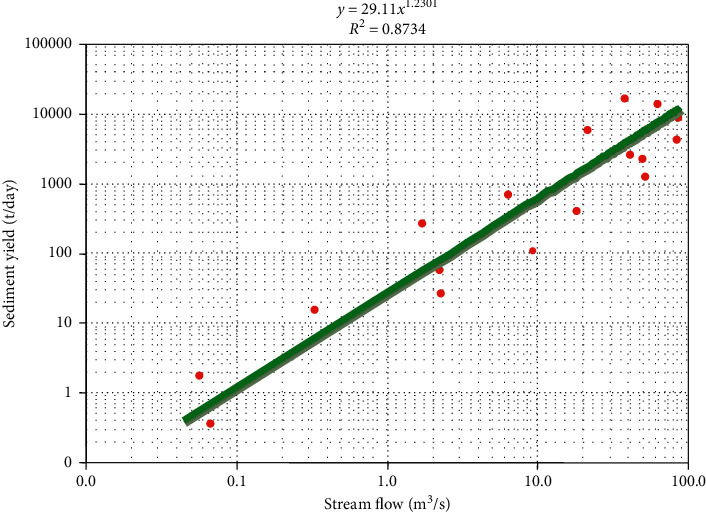
Sediment rating curve of Megech River near Azezo gauge station.

Figure 11 shows the sediment rating curve of Megech River near Azezo gauge station.

Using the rating curve equation, the continuous-time step suspended sediment load (t/day) values were generated from the daily based records of streamflow. But SWAT simulation is based on the total sediment load (suspended + bed load). Hence, considering the mountainous nature of Megech River and its three main tributaries directly joining the Megech dam reservoir while their flood velocities are high (higher than 3 m/s), it is appropriate to add bedload (sand, gravel, and boulder) transported by the river. However, there is no measured data on bedload material because direct measurement of bedload transport is very difficult and also inaccurate. But, in most rivers, bedload to suspended load ratio is in the range of 1 : 5–1 : 50. Therefore, it was decided that the bedload for the Megech watershed would be estimated as being 10% of the suspended load [16].

### 3.4. Modeling the Impact of Land Use Dynamics on Catchment Hydrology

Seven flow parameters, namely, soil evaporation compensation factor, alpha base flow recession constant (days), initial SCS CN-II value (%), available water capacity (mm water/mm soil), soil depth (mm), threshold depth of water required for return flow to occur (mm), and threshold depth of water required for evaporation to occur (mm) with a sensitivity class of high to medium, were selected for calibration of the 1998 LULC phase. Whereas one additional flow parameter, namely, effective channel hydraulic conductivity (mm/h), was found to be sensitive for the 2016 LULC phase.

As the default SWAT simulation of Megech dam watershed shows, there was a little discrepancy between measured data and simulated outputs. This may be resulted from the quality of measured weather and flow data used as an input to the model. Some of the stations had many missing weather data, which were left to be estimated and filled by the model's weather generator. Hence, using these estimated data may influence the simulation output. Besides, error in measurement of flow and weather data may be another reason for the slight variation between measured and simulated flows at peak and low flow seasons. To solve this mismatch between measured and simulated outputs, a combination of the automatic and manual calibration was performed until the simulated result coincides with the observed data. As it could be seen from figure, the simulated streamflow after calibration shows a good agreement with the observed dataset.

**Figure 12:**
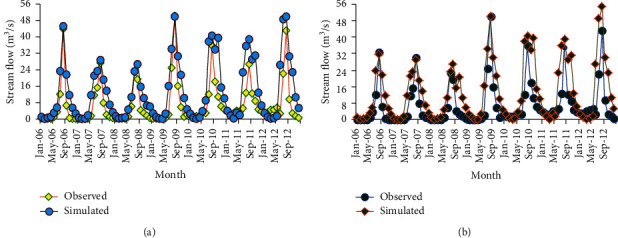
Stream flow comparison for calibration periods (a) 1998 LULC and (b) 2016 LULC.

Figures 12(a) and 12(b) show streamflow hydrograph of measured and simulated values for 1998 historical land use and 2016 recent land use, respectively.

Even though the pattern agreement was good for the simulated and calibrated model, the stream flow volume error was found to be slightly larger (−13.12 for 1998 LULC calibration and −11.05 for 2016 LULC calibration). The possible causes can be inefficient manual calibration, incorrect rainfall, and streamflow records, error in estimating missed flow, and precipitation data or it may be also due to the use of the LULC map out of the calibration period. On both land use phases, the model well senses the seasonal variability of streamflow. The model slightly shows a good response to extreme rainfall events (August 2006 and 2009) resulting in high runoff. Calibrated values of flow parameters for both LULC phases are shown in Tables 7 and 8.

**Figure 13:**
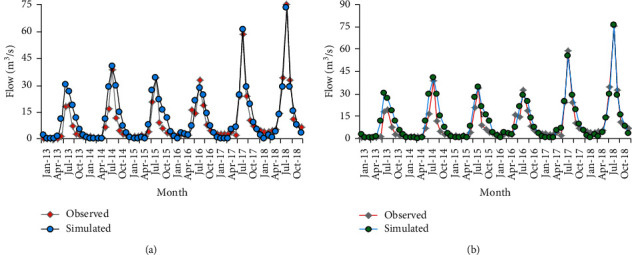
Stream flow comparison for validation periods (a) 1998 LULC and (b) 2016 LULC.

Validation of the model by using an independent set of measured flow data was carried out without further adjustment of the calibrated flow parameters.  Figure 13 shows the observed and simulated streamflow for the validation period.

The performance of the model was evaluated and gives a satisfactory value of R2 and ENS for both land use scenarios (Table 9).

Even though the (*R*^2^) and (ENS) value lies in an acceptable range, the relative volume error for both land use phases is somewhat large. Whereas the values of correlation coefficient (*R*^2^) and Nash and Sutcliffe simulation efficiency (ENS) are satisfactory indicators of goodness-of-fit between monthly measured and simulated stream flows in the validation period. The result also indirectly indicates that a model was calibrated well to simulate monthly stream flows adequately. Hence, it is possible to say that the SWAT model was successful to simulate realistic flow with a little deviation from observed stream volume for this particular research. The 18 years impact of land use dynamics on the mean annual catchment hydrology was quantified as shown in Table 10.

**Figure 14:**
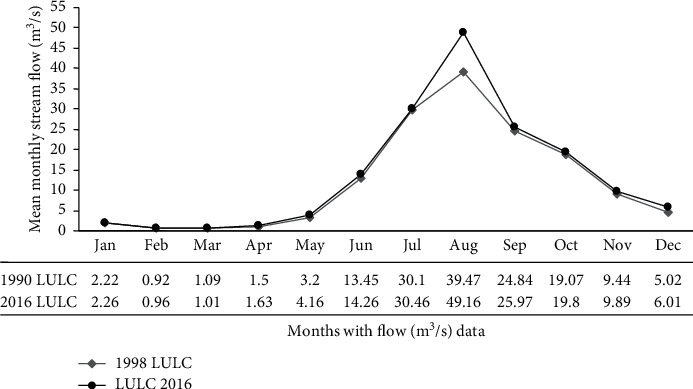
Simulated streamflow comparison under land use dynamics (2004–2018).

The catchment hydrology result indicated that the mean annual surface runoff for 2016 recent land cover was increased by 26.03% than the 1998 historical land cover. The mean annual flow of Megech stream was increased by 15.3 m^3^/s within 18 years. This implies that the 2016 LULC mean annual streamflow was increased by 10.2% than 1998 LULC. The details of the simulated monthly streamflow comparison between the two reference land uses are shown in Figure 14.

The monthly streamflow hydrograph indicated that the average mean monthly streamflow for 1998 LULC was 12.53 m^3^/sec and that of 2016 LULC was 13.79 m3/sec. With this flow pattern, the 2016 LULC average monthly streamflow was increased by 10.15% than 1998 LULC. The maximum flow percentage increment was observed during August which is 24.55% (from 49.16 m^3^/sec in 1998 LULC to 39.47 m^3^/sec in 2016 LULC).

**Figure 15:**
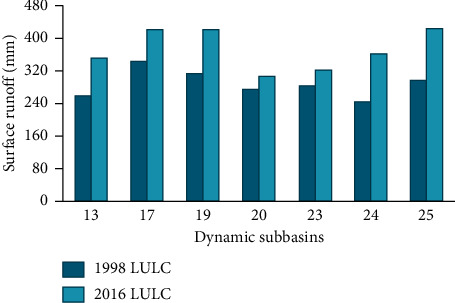
Comparison of simulated surface runoff (mm) for selected dynamic subbasins.

Comparisons were made between the two reference land uses to evaluate the impact of land use dynamics on the spatial distribution of simulated surface runoff. The dominant land cover changes were observed in the southern, southwestern, and central parts of the study area, which is coded in the figure as subbasins 13, 17, 19, 20, 23, 24, and 25. The tabular and graphical comparisons of surface runoff for selected dynamic subbasins are shown in Table 11 and Figure 15, respectively.

The dominant land covers in the above change sensitive subbasins were forest, grassland, and barelands in 1998 and had been changed to cultivated land and urban in 2016. Due to such land use changes, surface runoff increases by 37.24%, 23.93%, 35.19%, 12.06%, 14.97%, 49.67%, and 44.29% for subbasins 13, 17, 19, 20, 23, 24, and 25, respectively. Recently, subbasin 25 contributes the highest mean annual surface runoff (427.48 mm) to Megech stream flow, which was 296.26 mm during 1990. Whereas subbasins 17 and 19 take the second and third contributions, respectively. The overall summary of the subbasin level land use dynamics impact on catchment hydrology is shown in Table 12.

### 3.5. Modeling the Impact of Land Use Dynamics on Sediment Yield

Sensitivity analysis was carried out for 1998 historical land use and 2016 recent land use separately. Five sediment parameters (USLE support practice factor, average slope steepness (m/m), linear factor for channel sediment routing, available water capacity (mm water/mm soil), and exponential factor for channel sediment routing) with a sensitivity class of very high to medium were selected for calibration of the 1998 LULC phase. Whereas one additional parameter (soil albedo) was found to be sensitive for the 2016 LULC phase.

Unlike streamflow simulation, the mismatch gap between measured and simulated sediment yield was found to be large for default simulation. The possible cause for this variation might be the lack of enough measured sediment data used during sediment rating curve development as most of the sediment samples were not representatives of the whole simulation periods. The sediment yield calibration was carried out by varying sediment sensitive parameters iteratively within the allowable ranges until a satisfactory agreement between observed and simulated sediment yield was obtained. Tables 13 and 14 show the calibrated values of sediment parameters.

**Figure 16:**
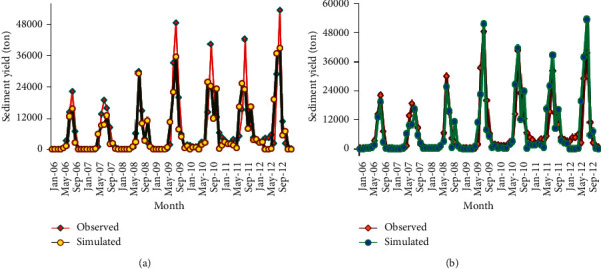
Sediment flow comparison for calibration periods (a) 1998 LULC and (b) 2016 LULC.

Last, monthly time step sediment yield hydrograph was developed to compare the observed and simulated sediment load values for the calibration period (2006–2012).  Figure 16 shows the sediment yield hydrograph of measured and simulated values.

As the sediment yield hydrograph of the calibration period shows the monthly observed and simulated sediment load matched well for the 2016 LULC phase. But the 1998 LULC phase sediment simulation result was found to be underestimated. This underestimation was likely observed on the peak sediment flow month (August). This might be due to the SWAT model characteristics to undermine the relatively low sediment yield of the 1998 LULC phase when it is compared with the 2016 LULC phase. Because the sediment load of 1998 LULC was relatively small, as it was compared with 2016 LULC, which is mainly caused by land use dynamics. Unlike peak sediment yield seasons, there was a good agreement between the measured and simulated sediment load for low sediment flow seasons (September–May).

Disparate from calibration, the sediment yield hydrograph of measured and simulated output during the validation period shows a good agreement for both reference land uses. This was mainly due to the availability of enough measured sediment samples taken during the validation period, which later used for sediment rating curve preparation. This ensures that the observed sediment load data used for model input during the validation period were more representative and a better approach to reality than the calibration period.

**Figure 17:**
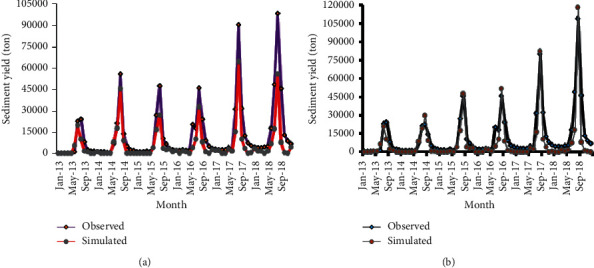
Sediment flow comparison for validation periods (a) 1998 LULC and (b) 2016 LULC.

The hydrographs of Figure 17 show that during the validation period, the model underestimates both the peak and low sediment yields for the 1998 LULC phase. Whereas the model slightly overestimated the peak sediment yields during August for the 2016 LULC phase. This might be due to the characteristics of the SWAT model to sense the increased sediment load due to land use changes. Because the land use change from 1998 to 2016 causes a significant increment of sediment load significantly as shown from the hydrographs.  Table 15 shows the performance measures of the model for both calibration and validation periods.

The mean annual sediment yield for 2016 land cover was increased by 33.3% than the 1998 historical land cover. 26.03% increment of surface runoff produced 33.33% sediment yield within 18 years difference. This shows how much the catchment currently becomes susceptible to erosion, which is due to the expansion of intensive agricultural practice. The 18 years impact of land use dynamics on the mean annual sediment yield was quantified as shown in Table 16.

**Figure 18:**
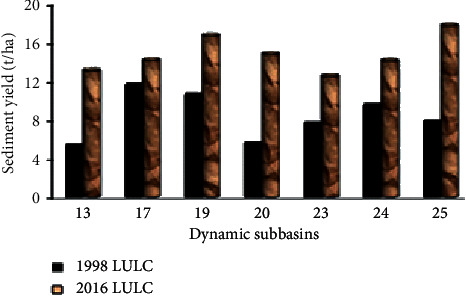
Sediment yield for selected dynamic subbasins.

Proportional to surface runoff equivalent remarks were also observed for the sediment yield pattern in subbasins 13, 17, 19, 20, 23, 24, and 25 where grassland, bareland, and forest land covers in 1998 have changed to cultivated and urban land in 2016 as shown in Figure 18 and Table 17.

The sediment yield were increased by 149.23%, 22.86%, 58.55%, 172.22%, 65.73%, 48.94%, and 128.7% for subbasins 13, 17, 19, 20, 23, 24, and 25, respectively. Recently, subbasin 25 contributes the highest mean annual sediment load (17.61 t/ha/year) to Megech dam reservoir. This subbasin was covered mostly with shrubland cover during 1998 and had been largely changed to cultivated land in 2016, whereas subbasin 19 (16.6 t/ha/year) and subbasin 17 (14.08 t/ha/year) contribute the second and third most sediment load to Megech dam reservoir, respectively. The overall summary of the subbasin level impact of land use dynamics on sediment production is shown in Table 18.

## 4. Conclusions

The analysis of land use dynamics between the two reference LULC (1998 LULC and 2016 LULC) indicated that upper Megech dam watershed had experienced a significant change in land use/land cover over the past 18 years. The main causes of this significant change were urbanization and expansion of the intensive agricultural practice in the area, which later results in rapid alteration of grassland, bareland, and plantation forest to cultivated land and urban. This was evident through 6.48% and 1.06% increment of cultivated land and urban land, respectively, in 18 years. In contrast, grassland, plantation forest, and bareland coverage were reduced by 6.09%, 0.94% (deforestation), and 1.02%, respectively. Keeping all other hydrometeorological variables constant and varying only the LULC map of upper Megech dam watershed result in a significant change in runoff and sediment yield. This was manifested by the increase in simulated mean annual surface runoff by 26.03%, mean annual streamflow by 10.2%, and mean annual sediment yield by 33.3% from 1990 LULC to 2016 LULC.

Megech dam design report shows that the dead storage capacity of the dam was designed by considering 11.7 t/ha/year sedimentation rates. But, due to land use dynamics, currently, 13.6 t/ha/year sediment is entering to Megech dam reservoir. To ensure the effective use of Megech dam reservoir, either appropriate measures on the catchment land use practice have to be taken or the dead storage design of the reservoir should be revised. The knowledge of how land use dynamics influence watershed hydrology enables local governments and policymakers to formulate and implement appropriate response strategies to minimize the undesirable effects of future land use/cover change. Therefore, having the knowledge and quantifying the impacts of land use dynamics on the hydrologic conditions and sediment production are helpful for optimal management of Megech dam watershed. This study also contributes a lot to identify the most dynamic and change sensitive subbasins within the watershed, so that the decision-makers might implement watershed management policies on such selected dynamic subbasins. Present, subbasins 13, 17, 19, 20, 23, 24, and 25 are found to be the major sources of sedimentation in Megech dam watershed. Hence, the local government should carry out catchment treatment on these dynamic and change sensitive subbasins, so that it can be possible to minimize undesirable future impacts on the reservoir.

Due to the difference in the scale, spatial resolution, and season of map preparation, securing different land use maps were the major constraints of this study. Hence, a comparison of only two reference land uses (1998 historical land use and 2016 recent land use) that have the same spatial resolution, scale, and season of preparation were made. Therefore, other researchers are recommended to make comparisons of more than two reference land uses for the future, so that it is possible to have an improved impact evaluation of land use dynamics on hydrology and sediment yield. Another major limitation during this research work was the lack of continuous measured suspended sediment data. Only a few sediment concentration measurements were available during different years. The best option for this problem was to generate the daily sediment data from sediment rating curves developed by using available measurements. Therefore, to get better-simulated sediment output that approaches to the actual measured data, responsible bodies are recommended to record frequent and reliable sediment data. Finally, this study considered only land use/land cover changes to compare the corresponding changes on hydrology and sediment yield. But other variables such as climate changes and management activities might have a significant impact on runoff and sediment yield of the research area. Hence, future researchers are highly recommended to consider the climate change impact on streamflow and sediment yield.

## Figures and Tables

**Table 1:** Area weightage of rainfall stations.

Rainfall stations	Elevation (m)	*X*-coordinate (m)	*Y*-coordinate (m)	Area weightage (*Ai*/*At*)	Mean annual RF (mm)
Gorgora	1830	315087	1354781	0.082914708	1085
Aykel	2254	289055	1387012	0.240640363	1628
Ambagiorgis	2900	348028	1411743	0.131562083	976
Chewahit	1925	307352	1364265	0.163125406	1020
Gondar	1973	329618	1384684	0.220645572	1103
Maksegnit	1912	342912	1369920	0.161111869	1096

**Table 2:** Slope class of Megech dam watershed (FAO, 2002).

Landforms	Slope class (%)	Area (ha)	Area coverage (%)
Flat	0–3	624.69	1.58
Undulating plains	3–8	5215.53	13.23
Rolling plains	8–15	8462.60	21.47
Hilly plains	15–30	14767.17	37.46
Mountains	>30	10349.21	26.25

**Table 3:** Spatial distribution of soil types within the watershed.

Soil type	SWAT_code	Area (ha)	Area coverage of the watershed (%)
Eutric Leptosols	ReLp	33250.10	84.35
Haplic Nitisols	RhNT	3733.00	9.47
Chromic Luvisols	RxLv	1486.10	3.77
Urban	U	409.96	1.04
Eutric Vertisols	AeVr	543.98	1.38

**Table 4:** Land use/land cover as reclassified by SWAT.

Original land use	SWAT code	Area (ha) for historical (1998 LULC)	Area (ha) for recent (2016 LULC)
Cultivated land	AGRC	23922.14	26478.21
Plantation forest	FRSE	723.54	354.31
Grassland	PAST	4502.82	2101.48
Shrubland	RNGB	7951.23	8151.54
Bareland	URLD	1412.15	1008.52
Urban	URHD	907.32	1325.14

**Table 5:** Calibration and validation periods for flow and sediment.

Types of simulation	Period of simulation	
	Historical land use(1998 LULC)	Recent land use (2016 LULC)
Flow calibration	2004–2012	2004–2012
Flow validation	2013–2018	2013–2018
Sediment calibration	2004–2012	2004–2012
Sediment validation	2013–2018	2013–2018

**Table 6:** Summary of spatial analysis result for land use dynamics.

Original land use	SWAT code	Area (ha)		Area coverage (%)		Dynamics (%)
		1998 LULC	2016 LULC	1998 LULC	2016 LULC	2016 LULC–1998 LULC
Cultivated land	AGRC	23922.14	26478.21	60.69	67.17	6.48
Plantation forest	FRSE	723.54	354.31	1.84	0.90	-0.94
Grass land	PAST	4502.82	2101.48	11.42	5.33	-6.09
Shrubland	RNGB	7951.23	8151.54	20.17	20.68	0.51
Bareland	URLD	1412.15	1008.52	3.58	2.56	-1.02
Urban	URHD	907.32	1325.14	2.30	3.36	1.06

**Table 7:** Calibrated values for 1998 historical LULC.

Rank	Flow parameters	Allowable range	Calibrated values
1	Gwqmn	0–1000	340
2	Alpha_Bf	0–1	0.13
3	Esco	0–1	0.83
4	Cn2	±25	+16%
5	Revapmn	0–1000	470
6	Sol_Awc	±25	+14%
7	Sol_Z	±25	9.3%

**Table 8:** Calibrated values for 2016 recent LULC.

Rank	Flow parameters	Allowable range	Calibrated values
1	Alpha_Bf	0–1	0.11
2	Gwqmn	0–1000	412
3	Ch_K2	0–1	0.51
4	Cn2	±25	+10%
5	Esco	0–1	0.63
6	Sol_Awc	±25	+5.6%
7	Sol_Z	±25	21%
8	Revapmn	0–1000	348

**Table 9:** Measures of model performance for flow.

Parameters	1998 historical LULC		2016 recent LULC	
	Calibration (2006–2012)	Validation (2013–2018)	Calibration (2006–2012)	Validation (2013–2018)
*R* ^2^	0.86	0.91	0.78	0.89
ENS	0.73	0.86	0.69	0.83
RVE	−13.12	−9.71	−11.05	−7.42

**Table 10:** Change in catchment hydrology parameters due to land use dynamics.

Hydrology parameter	1998 historical LULC	2016 historical LULC
Annual Sur_Runoff (mm)	251.3	316.7
Annual baseflow (mm)	516.3	504.3
Annual streamflow (m^3^/s)	150.3	165.6
Total water yield (mm)	767.6	821

**Table 11:** Simulated surface runoff (mm) for selected dynamic subbasins.

Dynamic subbasins	1998 LULC	2016 LULC	Change (mm)
13	256.15	351.53	95.38
17	341.95	423.79	81.84
19	312.41	422.36	109.95
20	272.88	305.80	32.92
23	282.55	324.86	42.31
24	242.17	362.46	120.29
25	296.26	427.48	131.22

**Table 12:** Summary of simulated mean annual runoff (mm) comparison at the subbasin level.

Subbasins	Area (km^2^)	1998 LULC	2016 LULC
1	14.198	251.08	270.67
2	18.899	189.15	262.28
3	3.2404	244.28	240.58
4	22.724	219.65	241.9
5	21.781	234.15	270.63
6	11.091	221.94	256.41
7	12.92	219.82	319.61
8	2.6224	307.63	308.31
9	6.1384	231.92	387.59
10	8.6188	231.66	338.34
11	13.078	202.93	250.68
12	18.707	195.17	222.2
13	8.3766	256.15	351.53
14	0.90197	243.94	271.23
15	9.0113	220.19	293.68
16	12.168	286.63	291.48
17	30.917	341.95	423.79
18	13.321	221.88	299.62
19	10.381	312.41	422.36
20	16.311	272.88	305.8
21	2.3885	246.83	363.25
22	3.4575	229.64	345.78
23	10.907	282.55	324.86
24	13.939	242.17	362.46
25	69.259	296.26	427.48
26	9.8381	291.31	283.81
27	20.478	256.85	358.68
28	3.7916	292.33	329.34
29	4.727	245.48	361.88

**Table 13:** Calibrated values for 1998 historical LULC.

Rank	Sediment parameters	Allowable range	Calibrated values
1	Usle_P	0–1	0.67
2	Slope	±25	+8%
3	Spcon	0.0001–0.01	0.0075
4	Sol_Awc	±25	+11%
5	Spexp	1–2	0.56

**Table 14:** Calibrated values for 2016 recent LULC.

Rank	Sediment parameters	Allowable range	Calibrated values
1	Usle_P	0–1	0.77
2	Slope	±25	+5%
3	Spcon	0.0001–0.01	0.0053
4	Sol_Awc	±25	+13%
5	Sol_Alb	±25	−2%
6	Spexp	1-2	1.26

**Table 15:** Measures of model performance for sediment.

Parameters	1998 historical LULC		2016 recent LULC	
	Calibration (2006–2012)	Validation (2013–2018)	Calibration (2006–2012)	Validation (2013–2018)
*R* ^2^	0.70	0.88	0.86	0.92
ENS	0.69	0.80	0.74	0.84
RVE	−12.87	−10.56	−8.23	−7.85

**Table 16:** Change in sediment yield due to land use dynamics.

Sediment parameter	1998 historical LULC	2016 historical LULC
Mean annual sediment yield (t/ha/year)	10.23	13.61

**Table 17:** Comparison of simulated sediment yield (t/ha/year) for selected dynamic subbasins.

Dynamic subbasins	1998 LULC	2016 LULC	Change (t/ha/year)
13	5.22	13.01	7.79
17	11.46	14.08	2.62
19	10.47	16.60	6.13
20	5.40	14.70	9.30
23	7.50	12.43	4.93
24	9.42	14.03	4.61
25	7.70	17.61	9.91

**Table 18:** Summary of simulated mean annual sediment yield (t/ha/year) comparison at the subbasin level.

Subbasins	Area (km^2^)	1998 LULC	2016 LULC
1	14.198	9.99	11.3
2	18.899	11.29	11.53
3	3.2404	8.62	8.1
4	22.724	11.9	12.4
5	21.781	9.29	9.58
6	11.091	7.53	10.56
7	12.92	16.53	17.52
8	2.6224	10.27	9.5
9	6.1384	7.7	14.34
10	8.6188	13.73	16.58
11	13.078	11.68	13.62
12	18.707	11.9	14.69
13	8.3766	5.22	13.01
14	0.90197	9.78	16.31
15	9.0113	13.57	14.44
16	12.168	9.25	8.72
17	30.917	11.46	14.08
18	13.321	14.71	17.14
19	10.381	10.47	16.6
20	16.311	5.4	14.7
21	2.3885	11.17	16.62
22	3.4575	9.53	14.4
23	10.907	7.5.	12.43
24	13.939	9.42	14.03
25	69.259	7.7	17.61
26	9.8381	8.68	7.95
27	20.478	11.26	16.43
28	3.7916	8.48	13.16
29	4.727	12.55	17.3

## Data Availability

The spatial data used in this study were obtained from the Ministry of Agriculture and Rural Development (MoARD) of Ethiopia. The hydrological and meteorological data can be found from the Ministry of Water, Irrigation, and Energy (MoWIE) and the National Meteorological Service Agency (NMA) of Ethiopia, respectively. These data are also made available from the corresponding author upon request.

## Supplementary Materials

Supplementary Materials Appendix 1: mean annual rainfall of selected gauge stations (National Meteorological Service Agency of Ethiopia, 2016). Appendix 2: selected weather stations and their respective locations (National Meteorological Service Agency of Ethiopia, 2016). Appendix 3: values of soil parameters used for the study area (Ministry of Agriculture and Rural Development of Ethiopia, 2017). Appendices 4: definition of weather generator statistical parameters (National Meteorological Service Agency of Ethiopia, 2016). Appendix 5: weather generator statistics and probability values of Gondar Station (National Meteorological Service Agency of Ethiopia, 2016).
