# Supplementary material for: Modeling Impact of Land Use Dynamics on Hydrology and Sedimentation of Megech Dam Watershed, Ethiopia
Source: ScientificWorldJournal. 2020 Oct 24;2020:6530278. doi: 10.1155/2020/6530278 (PMC7604611; doi:10.1155/2020/6530278)
Supplement: Supplementary Materials — Appendix 1: mean annual rainfall of selected gauge stations (National Meteorological Service Agency of Ethiopia, 2016). Appendix 2: selected weather stations and their respective locations (National Meteorological Service Agency of Ethiopia, 2016). Appendix 3: values of soil parameters used for the study area (Ministry of Agriculture and Rural Development of Ethiopia, 2017). Appendices 4: definition of weather generator statistical parameters (National Meteorological Service Agency of Ethiopia, 2016). Appendix 5: weather generator statistics and probability values of Gondar Station (National Meteorological Service Agency of Ethiopia, 2016). [file 6530278.f1.pdf]

Appendix 1: Mean Annual Rainfall of selected gauge stations

Stations	mean annual RF (mm)	class
Aykel	1628	class-1
Gonder	1103	class-1
Amba giorgis	976	class3
Maksegnit	1096	class-3
Chewahit	1020	class-4
Gorgora	1085	class-4

Appendix 2: Selected Weather Stations and their Respective Locations

Station	latitude (degree)	longitude (degree)	Elevation a.s.l (m)	X-Coordinate (m)	Y-Coordinate (m)
**Gorgora**	121500	371800	1830	315087	1354781
**Aykel**	123223	370331	2254	289055	1387012
**Ambagiorgis**	124600	373600	2900	348028	1411743
**Chewahit**	122007	371342	1925	307352	1364265
**Gonder**	123116	372555	1973	329618	1384684
**Maksegnit**	122318	373318	1912	342912	1369920

Appendix 3: Values of Soil parameters used for the study area

**SNAM**	**Haplic Nitisols**				**Chromic Luvisols**						
NLAYERS	4				7						
HYDGRP	C				B						
SOL_ZMX	2000				1800						
ANION_EXCL	0.01				0.01						
SOL_CRK	0.01				0.01						
TEXTURE	SICL	CL	SC	C	SIL	SIL	SIL	SILL	SIL	SIL	SIL
SOL_Z	200	900	1000	2000	200	260	460	650	950	1350	1800
SOL_BD	1.1	1.27	1.28	1.22	1.45	1.46	1.45	1.49	1.5	1.49	1.47
SOL_AWC	0.11	0.11	0.1	0.11	0.12	0.11	0.1	0.1	0.1	0.1	0.1
SOL_K	4.34	4.54	5.16	4.24	7	37.2	34.8	33.6	36	36	36
SOL_CBN	2	1.5	1.3	0.5	0.5	0.3	0.21	0.2	0.2	0.12	0.1
CLAY	50	23	60	71	25	14	19	22	17	17	16
SILT	33	50	25	20	31	66	59	56	57	57	59
SAND	17	27	15	9	44	20	22	22	26	26	25
ROCK	5	0	0	0.13	0.01	0	0	0	0	0	0
SOL_ALB	0.13	0.13	0.13	0.22	0.13	0.13	0.13	0.13	0.1	0.13	0.13
USLE_K	0.22	0.22	0.22	0.22	0.3	0.3	0.3	0.3	0.3	0.3	0.3

SNAM	Eutric Leptosols		Urban			Eutric Vertisols						
NLAYERS	2		3			7						
HYDGRP	C		D			D						
SOL_ZMX	650		1500			2422						
ANION_EXCL	0.01		0.01			0.01						
SOL_CRK	0.01		0.01			0.03						
TEXTURE	CL	SCL	SIL	SICL	SIC	CL	CN	SIL	CNV	FSL	UWB	C
SOL_Z	200	650	280	737	1500	250	363	847	1029	1392	1635	2422
SOL_BD	1.1	1.13	1.13	1.45	1.6	1.08	1.27	1.28	1.22	1.13	1.1	1.1
SOL_AWC	0.13	0.1	0.22	0.11	0.16	0.12	0.11	0.1	0.1	0.1	0.11	0.09
SOL_K	25	13	6.6	10.7	5.33	6.8	4.54	5.16	4.24	4.34	4.24	4.04
SOL_CBN	2	1.1	1.49	1.02	0.15	1.6	1.37	1.41	0.88	1.17	1.24	0.34
CLAY	50	66	26.5	37.5	45	53.6	60.6	62.6	62.8	62.6	60	63.6
SILT	34	14	20	55	47.6	25.7	18.6	17	8.4	9.4	12.7	16.6
SAND	17	20	23.5	7.47	7.37	20.7	20.8	20.4	28.8	28	27.3	19.8
ROCK	5	0.01	1.09	0.08	0	0	0	0	0	0	0	0
SOL_ALB	0.13	0.13	0.13	0.03	0.17	0.13	0.09	0.09	0.09	0.09	0.09	0.09
USLE_K	0.22	0.22	0.32	0.49	0.49	0.23	0.2	0.2	0.2	0.2	0.2	0.2

Appendices 4: Definition of Weather Generator statistical parameters

**tmpmx**	Average or mean daily maximum air temperature for month (^oC)
**tmpmn**	Average or mean daily minimum air temperature for month (^oC)
**tmpstdmx**	Standard deviation for daily maximum air temperature in month(^oC)
**tmpstdmn**	Standard deviation for daily minimum air temperature in month(^oC)
**pcpmm**	Average or mean total monthly precipitation (mm H2O)
**pcpstd**	Standard deviation for daily for daily precipitation in month(mmH2O/day)
**pcpskw**	Skew coefficient for daily precipitati on in month
**pr_w(1)**	Probability of a wet day following a dry day in month
**pr_w(2)**	Probability of a wet day following a wet day in month
**pcpd**	Average number of days of precipitation in month
**solarav**	Average daily solar radiation for month(MJ/m2/day)
**dewpt**	Average daily dew point temperature in month(^oC)
**windav**	Average daily wind speed in month(m/s)

Appendix 5: Weather generator statistics and probability values of Gonder Station

	Jan	Feb	Mar	Apr	May	Jun	Jul	Aug	Sep	Oct	Nov	Dec
**tmpmx**	28.21	29.83	30.28	30.38	29.17	25.70	23.15	23.36	25.45	26.78	27.53	27.71
**tmpmn**	11.41	12.90	13.99	15.00	14.69	13.70	13.07	13.03	12.45	12.08	11.78	11.42
**tmpstdmx**	1.35	1.48	1.99	2.03	2.36	2.04	1.57	1.53	1.51	1.36	1.18	0.96
**tmpstdmn**	2.40	2.76	3.07	3.12	3.50	2.65	2.62	2.49	2.53	2.55	2.58	2.26
**pcpmm**	3.65	3.11	14.18	31.67	88.26	191.86	329.81	318.89	116.46	88.16	21.21	8.50
**pcpstd**	1.15	0.87	2.46	3.41	6.67	10.50	11.69	11.38	6.97	7.93	3.12	2.06
**pcpskw**	12.59	10.78	11.74	5.77	3.87	3.02	2.10	2.02	2.84	4.45	6.82	11.57
**pr_w(1)**	0.02	0.03	0.09	0.16	0.28	0.59	0.91	0.84	0.42	0.25	0.09	0.04
**pr_w(2)**	0.25	0.20	0.31	0.49	0.58	0.73	0.92	0.90	0.59	0.46	0.36	0.32
**pcpd**	0.76	0.95	3.52	6.86	12.33	20.43	28.38	27.71	15.71	10.14	3.81	1.62
**solarav**	9.34	9.08	7.95	7.72	7.23	4.78	4.42	5.14	6.99	7.75	8.35	9.02
**dewpt**	10.20	9.76	10.63	11.25	13.65	16.20	16.92	16.79	16.53	14.68	12.56	10.79
**windav**	1.51	1.68	1.73	1.73	1.73	1.72	1.30	1.26	1.26	1.22	1.24	1.41
